# Tall fescue sward structure affects the grazing process of sheep

**DOI:** 10.1038/s41598-020-68827-0

**Published:** 2020-07-16

**Authors:** Leonardo Silvestri Szymczak, Anibal de Moraes, Reuben Mark Sulc, Alda Lucia Gomes Monteiro, Claudete R. Lang, Renata Francieli Moraes, Delma Fabiola Ferreira da Silva, Carolina Bremm, Paulo César de Faccio Carvalho

**Affiliations:** 1 Department of Crop Production and Protection, Federal University of Paraná, Rua dos Funcionários 1540, Curitiba, Paraná 80035-050 Brazil; 2 Department of Horticulture and Crop Science, The Ohio State University, 2021 Coffey Road, Columbus, OH 43210-1086 USA; 3 Departament of Animal Science, Federal University of Paraná, Rua dos Funcionários 1540, Curitiba, Paraná 80035-050 Brazil; 4 Department of Agricultural Diagnosis and Research, Secretary of Agriculture, Livestock and Irrigation of Rio Grande Do Sul, Rua Gonçalves Dias 570, Porto Alegre, Rio Grande do Sul 90130060 Brazil; 5 Department of Forage Plants and Agrometeorology, Federal University of Rio Grande Do Sul, Av. Bento Gonçalves 7712, Porto Alegre, Rio Grande do Sul 91540-000 Brazil

**Keywords:** Behavioural ecology, Grassland ecology, Agroecology

## Abstract

The study of factors influencing animal intake can provide a better understanding of the dynamics of the pasture ecosystem and serve as a basis for managing livestock in a more efficient way. We measured different sward surface heights of tall fescue in the process of short-term intake rate of sheep. There was a significant effect of sward surface height on herbage mass (*P* < 0.001), leaf lamina mass (*P* < 0.001), other species mass (*P* = 0.02), bite mass (*P* = 0.01) and short-term intake rate (*P* = 0.03) of sheep. There was a quadratic fit between time per bite and bite mass (*P* = 0.006). Multivariate analysis showed that the short-term intake rate and bite mass were positively correlated (r = 0.97), bite rate and total jaw movement rate were positively correlated but both were negatively correlated with time per bite. The sward surface height of tall fescue corresponding to the maximum short-term herbage intake rate was 22.3 cm. The underlying processes were driven by the bite mass, which was influenced by the leaf lamina bulk density and its consequences upon time per bite. This sward surface height can be adopted as a pre-grazing target for rotational stocking systems to optimize sheep nutrition on pastures.

## Introduction

In recent years alternative management of agricultural systems have been sought that support the principles of eco-efficiency^1–3^. Carvalho et al.^4^ and Laca et al.^5^ presented a perspective on precision livestock management, which is based on productive efficiency with environmental responsibility. A way to achieve these goals is through the understanding and mimicry of natural ecological processes^6^.

For millions of years herbivores and forage plants have been in a co-evolutionary pathway. Foraging strategies were developed to optimize herbage intake and meet the requirements for quantity and quality of feed that allow performance of other daily activities. In contrast, forage plants have developed morphological structures that restrict grazing in order to favor the development, growth and preservation of the species^7–10^.

The intake rate represents the consumption of forage per unit of time and is considered a fundamental component of the ingestive behaviour of grazing animals, being the product of bite mass and bite rate. In this context, the bite can be considered as the first scale of the grazing process^11–14^ and therefore, is under direct influence of the sward structure^8^.

Structural characteristics of the forage sward can stimulate, inhibit or limit the ingestive behaviour of animals^15–17^. These structural variables include leaf length and shape, cuticle thickness, stem physical properties, tiller density, proportion of senescent material, proportion and quantity of leaf blades, all of which are dependent on the species, growth habit, height, morphogenic characteristics, life cycle and longevity of the forage plant^17–24^. Thus, in the context of competition strategy at the plant–animal interface, the animals adapt to changes found in the pasture at the time of grazing, which promote behavioural changes, such as altering the pattern of displacement, food selection, the ratio of mass acquired and rate of harvest by animals, and mandibular and non-mandibular movements^25–28^.

The ingestive behaviour of animals has great relevance because it determines the daily nutrient intake and animal performance, as well as the location and intensity of the animal’s impact on the vegetation as well as the soil. Thus, a better understanding of ingestive behaviour can lead to a better understanding of the dynamics of the pasture ecosystem, providing the basis for more efficient management of animals and forage plants^14,19,29^.

In this experiment we tested the hypothesis that short-term intake rate (STIR) in sheep is maximized with intermediate tall fescue sward structures, expressed in sward surface height (SSH), and that both lower and higher SSH results in a decrease in bite mass. The specific objective of this study was to evaluate different SSH of a temperate perennial forage (*S. arundinaceus* [Schreb.] Dumort cv. Aurora), in the process of STIR of sheep, from a perspective of optimization of pasture management.

## Results

The actual pre- and post-grazing SSH increased linearly with expected SSH (treatments). There was no significant difference between pre- and post-grazing SSH for each treatment (*P* = 0.19 for 14 cm, *P* = 0.29 for 17 cm, *P* = 0.15 for 20 cm, *P* = 0.10 for 23 cm and *P* = 0.78 for 26 cm) with each grazing test period, so the average values between pre- and post-grazing were used for forage variables. The actual pre-grazing SSH were very similar to the intended SSH (Table 1).

**Table 1:** Actual sward surface heights pre- and post-grazing (SSH, cm), total herbage mass (HM, kg DM ha^−1^), leaf lamina mass (LLM, kg DM ha^−1^), pseudo-stem + sheath mass (PSM, kg DM ha^−1^), senescent mass (SM, kg DM ha^−1^) and other species mass (OSM, kg DM ha^−1^) as a function of intended swards surface heights (cm) of *Schedonorus arundinaceus* [Schreb.] Dumort (tall fescue).

	Sward surface height (cm)					SEM	*P* value
	14	17	20	23	26		
SSH pre-*	14.2^e^	17.3^d^	19.7^c^	22.8^b^	25.9^a^	0.12	0.000
SSH post-	14.0^e^	17.0^d^	19.6^c^	22.7^b^	25.9^a^	0.12	0.000
HM	1825^b^	1956^b^	2176^ab^	2158^ab^	2511^a^	59.1	0.000
LLM	1046^c^	1258^bc^	1387^b^	1333^b^	1659^a^	34.6	0.000
PSM	454	421	442	444	510	17.3	0.785
SM	377	278	347.69	381	342	16.5	0.743
OSM	17^b^	48^ab^	37^ab^	31^ab^	71^a^	5.0	0.020

*SEM* standard error of mean.

^a-e^Means within a row with different superscripts differ (*P* < 0.05) by Tukey’s test.

*There was no significant difference between pre- and post-grazing SSH for each treatment (*P* = 0.19 for 14 cm, *P* = 0.29 for 17 cm, *P* = 0.15 for 20 cm, *P* = 0.10 for 23 cm and *P* = 0.78 for 26 cm).

There was a significant effect of SSH treatment on HM (*P* < 0.001), LLM (*P* < 0.001) and OSM (*P* = 0.02) (Table 1). There was a linear relationship between the SSH and the LLM and HM. There was no significant effect of the SSH on the PSM (*P* = 0.78) and SM (*P* = 0.74), so the increase of HM is related to the increase of LLM.

**Figure 1:**
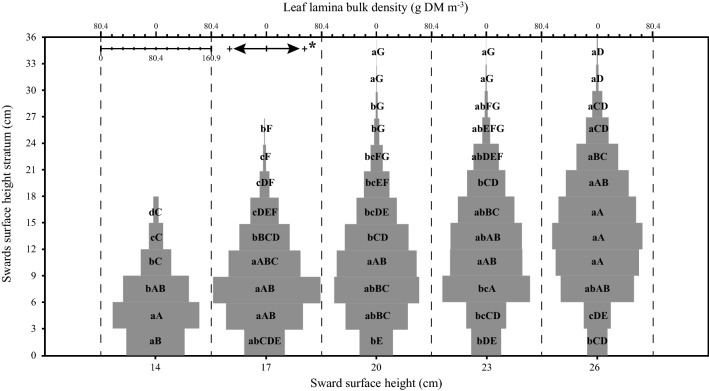
Leaf lamina bulk density by sward strata as a function of sward surface height (SSH) of *Schedonorus arundinaceus* [Schreb.] Dumort (tall fescue). *Total values of leaf lamina bulk density at each SSH correspond to the sum of the left and right sides. (**a)**–(**d)** The average values within a row with different lowercase letters differ (*P* < 0.05) by Tukey’s test. **(A)**–**(G)** The average values within a column with different uppercase letter differ (*P* < 0.05) by Tukey’s test. *P* significance level: SSH *P* < 0.001; Strata *P* < 0.001; SSH *×* Strata *P* < 0.001.

**Figure 2:**
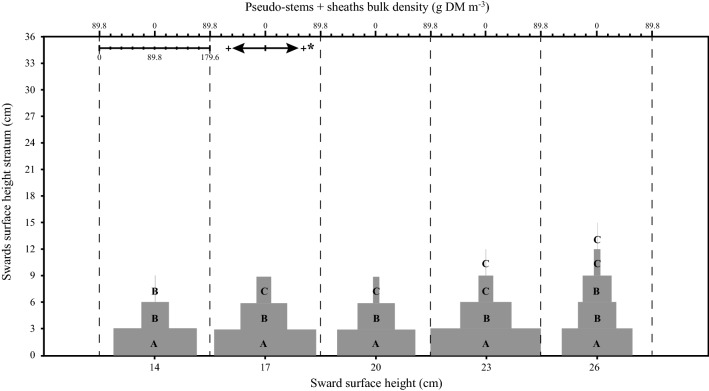
Pseudo-stem + sheath bulk density by sward strata as a function of sward surface height (SSH) of *Schedonorus arundinaceus* [Schreb.] Dumort (tall fescue). *Total values of pseudo-stem + sheath bulk density at each SSH correspond to the sum of the left and right sides. **(A)–(C)** The average values within a column with different uppercase letter differ (*P* < 0.05) by Tukey’s test. *P* significance level: SSH *P* = 0.349; Strata *P* < 0.001; SSH *×* Strata *P* = 0.366.

**Figure 3:**
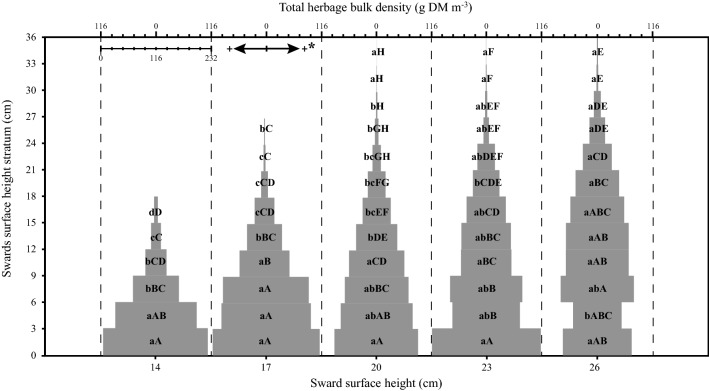
Total herbage bulk density by sward strata as a function of sward surface height (SSH) of *Schedonorus arundinaceus* [Schreb.] Dumort (tall fescue). *Total values of total herbage bulk density at each SSH correspond to the sum of the left and right sides. **(a)**–**(d)** The average values within a row with different lowercase letters differ (*P* < 0.05) by Tukey’s test. **(A)**–**(H)** The average values within a column with different uppercase letter differ (*P* < 0.05) by Tukey’s test. *P* significance level: SSH *P* < 0.001; Strata *P* < 0.001; SSH *×* Strata *P* = 0.05.

**Figure 4:**
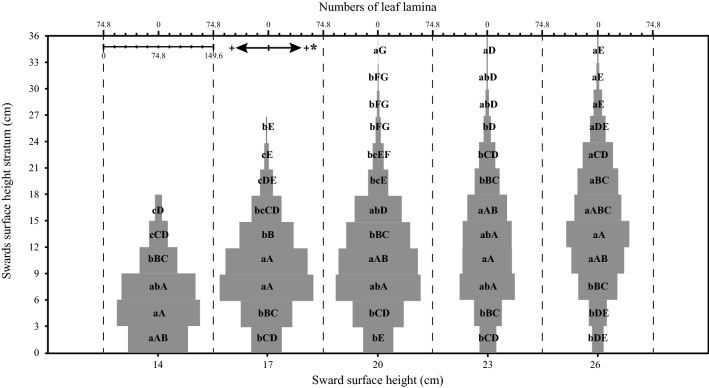
Numbers of leaf lamina by sward strata as a function of sward surface height (SSH) of *Schedonorus arundinaceus* [Schreb.] Dumort (tall fescue). *Total values of number of leaf lamina at each SSH correspond to the sum of the left and right sides. **(a)**–**(c)** The average values within a row with different lowercase letters differ (*P* < 0.05) by Tukey’s test. **(A)**–**(G)** The average values within a column with different uppercase letter differ (*P* < 0.05) by Tukey’s test. *P* significance level: SSH *P* < 0.001; Strata *P* < 0.001; SSH *×* Strata *P* < 0.001.

A significant SSH × stratum interaction was found for leaf lamina bulk density (*P* < 0.001), herbage total bulk density (*P* = 0.05) and number of leaf lamina (*P* < 0.001). For the pseudo-stem + sheath bulk density, there was a significant effect only between strata (*P* < 0.001). The highest leaf lamina bulk density occurred in stratum 3–6 cm for SSH of 14, 6–9 cm for SSH of 17, 20 and 23 cm and 9–18 cm for SSH of 26. As there was an increase in the SSH pre-grazing, there was also a linear increase in the leaf lamina bulk density (Fig. 1). The pseudo-stem + sheath bulk density was higher in the first stratum, decreasing in the upper strata (Fig. 2). The highest herbage total bulk density occurred in the stratum 0–6 cm for 14 and 20 SSH, 0–9 cm for 17 SSH, 0–3 for 23 SSH and 0–18 cm in the 26 SSH (Fig. 3). With the increase of the SSH pre-grazing, there was also an increase in herbage total bulk density in the upper strata (Fig. 3). The highest number of leaf lamina occurred in the stratum 3–9 cm for 14 SSH, 6–12 cm for 17 and 20 SSH, 6–18 cm for 23 SSH and 9–18 cm for the 26 SSH, consequently having a reduction of leaf number in the upper and lower strata (Fig. 4). During the grazing tests, the presence of other species was verified, with a larger mass of these at SSH of 26 and a smaller at SSH of 14 (Table 1). The proportional mass of other species in all treatments was less than 2.85% (Table 1). The species found were *Trifolium repens* L. (seedling stage), *Vicia sativa* L. (seedling stage), *Plantago tomentosa* Lam. (vegetative stage), *Oxalis corniculata* L. (vegetative stage), *Artemisia verlotorum* Lamotte (vegetative stage) e *Cynara cardunculus* L. (vegetative stage).

**Figure 5:**
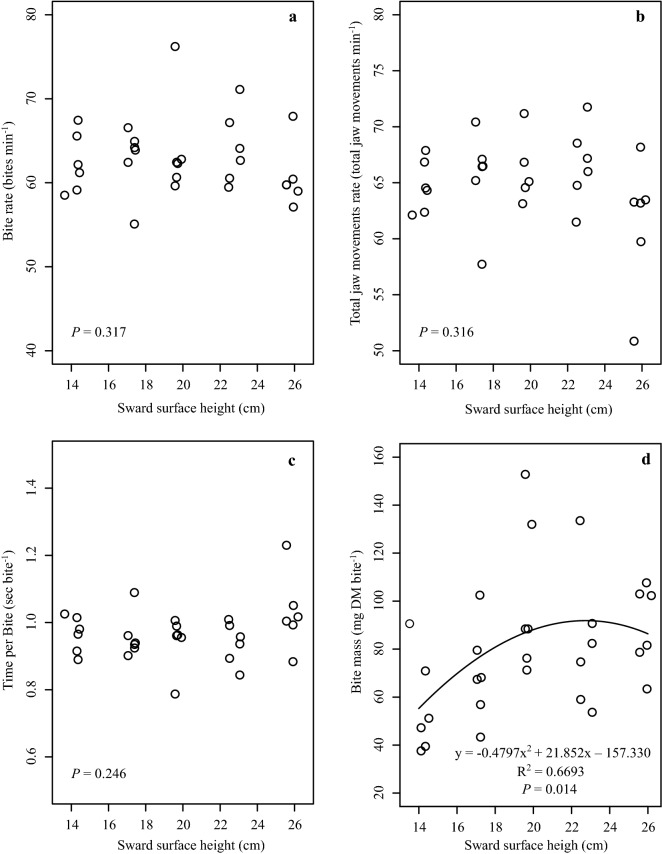
Bite rate **(a)**, total jaw movements rate **(b)**, time per bite** (c)** and bite mass **(d)** of sheep as a function of different *Schedonorus arundinaceus* [Schreb.] Dumort. (tall fescue) sward surface heights (SSH).

**Figure 6:**
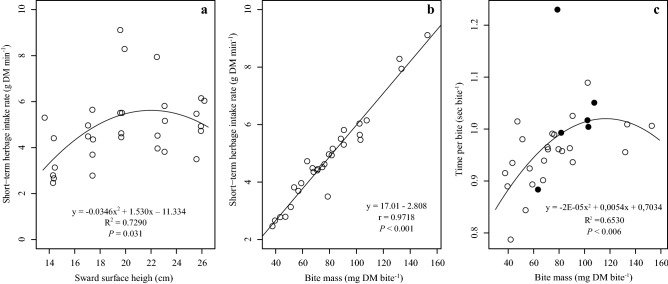
Short-term herbage intake rate as a function of sward surface height **(a)**, relationship between short-term herbage intake rate and bite mass **(b)** and relationship between time per bite and bite mass **(c)**—black circles represent animals under the effect of a sward surface height of 26 cm) of sheep in a *Schedonorus arundinaceus* [Schreb.] Dumort (tall fescue) pasture.

No effect was observed on the BR (*P* = 0.31), TJMR (*P* = 0.31) and TB (*P* = 0.24) as a function of SSH (Fig. 5a, b and c respectively). There was a significant effect of SSH of tall fescue on the BM (*P* = 0.01, Fig. 5d) and STIR (*P* = 0.03, Fig. 6a). There was a quadratic fit between STIR and SSH, with STIR increasing up to SSH of 22.3 cm (y = 5.61 g DM min^-1^; Fig. 6a) and then decreasing at greater SSH. The model that was best suited for BM as a function of SSH was also quadratic, with increasing BM up to 22.8 cm (y = 91.52 mg DM bite^-1^; Fig. 5d). A high correlation of 0.97 (*P* < 0.001) was also observed between STIR and BM variables (Fig. 6b). There was a quadratic fit between TB and MB (*P* = 0.006), with TB increasing up to BM of 135 mg DM bite^-1^ and then decreasing at greater BM (Fig. 6c).

**Figure 7:**
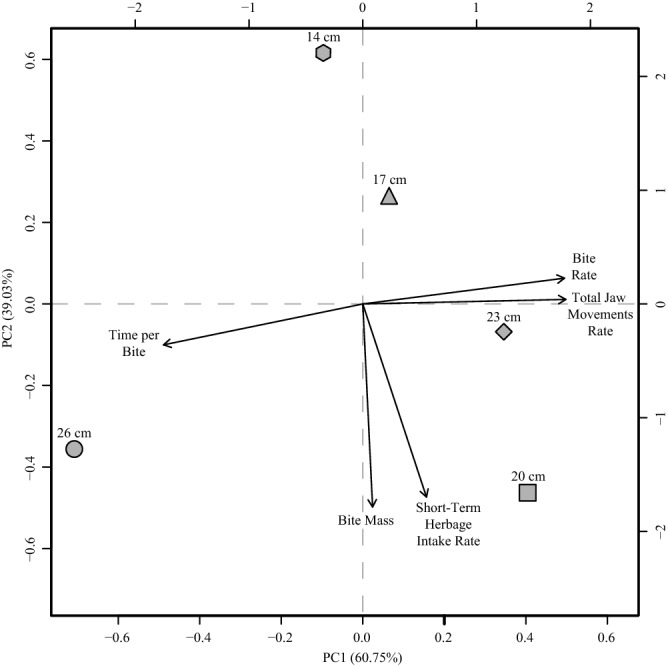
Principal component analysis of short-time herbage mass, bite mass, time per bite, bite rate and total jaw movements rate as a function of different *Schedonorus arundinaceus* [Schreb.] Dumort. (tall fescue) sward surface height (SSH). The two first dimensions explained 99.78% of the variability. The arrows within the graph represents distribution of variables and shows correlations between the different SSH and behaviour intake of sheep. The geometric shapes represent the mean values of SSH (14, 17, 20, 23 and 26 cm).

Multivariate analysis (Fig. 7) showed that the STIR and BM were positively correlated (r = 0.97). Bite Rate and TJMR were also positively correlated; however, both were negatively correlated with TB. The individual observations suggest that higher mean values for STIR and BM were obtained at SSH of 20 cm, while higher mean values for TB were obtained at SSH of 26 cm (Fig. 7).

## Discussion

The reduction of pre-grazing SSH by the animals during the grazing tests, as measured by the post-grazing SSH, did not exceed 5% (Table 1). This indicates that intended SSH were available to the animals from the beginning to the end of the grazing test.

The STIR response to SSH was a direct outcome from BM, since BR and TB were not affected by SSH. According to Baumont et al.^30^, sheep have exclusive prehension and mastication jaw movements, so TB would be a sum of a constant prehension time and a mastication time that increases linearly with BM.

Mezzalira et al.^22,31^ also registered similar results of STIR being affected by SSH when evaluating the contrasting sward structures of *Cynodon* sp. and *Avena strigose* Schreb. Ungar et al.^32^ and Laca et al.^33^ stated that STIR in homogeneous swards can be explained mainly by BM (Figs. 6B and 7). Thus, interpreting BM behaviour is fundamental for explaining STIR.

The ascendant part of the BM model is mechanistically rationalizable. BM is dependent on the bite volume (defined by bite depth and bite area) and the herbage bulk density of the forage^12,34,35^. At lower SSH the bite volume is commonly restricted by bite depth (e.g. Gregorini et al.^36^). As SSH increases from 14 to 22 cm, BM also increases mainly as a function of increasing bite depth and leaf lamina bulk density in the grazing horizon. This is the same response reported by Amaral et al.^17^ and Fonseca et al.^28^. However, BM reached the maximum level at 22.8 cm and did not increase at higher SSH, even though bite depth was expected to increase linearly in response to SSH^29^. This curvilinear response in BM is considered classic in sheep studies^14^, which led us to consider the asymptotic part of the BM model and the reason for this behaviour.

Bite depth can be depressed when high tensile-resisting stems act as a barrier to bite formation, as reported by Benvenutti et al.^37,38^. However, the depth for a bite free of the horizon of the pseudo-stem + sheath (grazing horizon) was 9, 18, 27, 24, and 21 cm for the SSH of 14, 17, 20, 23 and 26 cm, respectively (Figs. 1 and 2). Therefore, reduction of bite depth probably did not affect the response of BM and STIR to SSH. Barre^18^ found that shorter leaf lamina of perennial ryegrass was important in reducing the STIR of dairy cows. This may explain the lower STIR at SSH less than 22 cm in our study, as the length of the leaf lamina increased as the SSH increased (occupation by the leaf lamina of the upper strata of the sward at SSH above 20 cm) (Fig. 4). Sward architecture affecting leaf distribution in the upper layers of taller swards would have played a role, in addition to bite area constraints. Pretorius et al.^39^ argued that geometric principles, for both plant and animal, can explain BM affecting STIR. Spatial distribution of forage resources is driven by the plant thinning law, where the number of plants per unit space decreases at a rate of ¾ power as plant mass increases. So, the dilution of plant parts in space as its mass increases would have consequences to herbivore foraging. Our LLM data in the upper layers of the sward suggest that the leaf content in the bite volume could explain BM saturation, resulting in the asymptotic model for STIR (Fig. 6).

Using realistic three-dimensional (3-D) modeling studies of perennial ryegrass and tall fescue swards, Verdenal et al.^40^ and Sonohat et al.^41^ reported the increasing spatial distribution of the leaf blades in the upper strata of the sward with increasing SSH, resulting in a loss of verticality of leaf blades. As a consequence of this greater dispersion of leaf lamina, the animals are less likely to ingest large amounts of leaves in a single bite. Thus, the BM was likely reduced due to the lower accessibility of the leaf lamina mass, even though there was an increase in the leaf lamina bulk density in the higher SSH. Therefore, as the SSH approaches 23 cm, the increase in leaf lamina mass in the upper strata did not reflect an increase in sheep bite mass, altering the relationship between the availability (plant) and the acquisition capacity (animal) of the preferred item (leaf lamina).

The increasing distance of the plant organs (divarication) is considered by Greenwood and Atkinson^42^, McQueen^43^ and Bond et al.^44^ as an ecological strategy of evolutionary defense of trees and shrubs against grazing, limiting the bite mass of animals. We are not aware of published reports that discuss the evidence of divarication in grass species within the same context. However, empirical observations suggest that this same process occurs between domestic herbivores and grasses. In the case of sheep, this phenomenon can be very important due to the morphology of the mouth and the style of feeding, i.e. small opening width of the mandible and use of the lips and teeth to perform the bite. According to Shipley^8^ the interaction between the morphology of the mouth and the structure of plants together determine the mass of the bite that an animal can obtain. Thus, bite mass and volume have a prominent role in intra- and interspecific interactions in herbivorous communities and in pasture landscapes. At the SSH of 26 cm there may have been a need for more interaction time of the animal with the sward in the formation of bites, such as the positioning of the head and the choice of the location of the bite (Fig. 7). This can be interpreted in Fig. 6c, where the bites with intermediate mass (between 60 and 100 mg DM bite^−1^) and average TB greater than 1 s bite^−1^ are for SSH of 26 cm, compared with 0.96, 0.95, 0.94 and 0.93 s bite^−1^ for SSH of 14, 17, 20 and 23 cm, respectively.

According to Black and Kenney^34^, sheep tend to seek pasture structures that maximize the speed of ingestion. According to Baumont et al.^30^, the strategy of exploitation by the animals in horizons of the sward corresponds to a strategy of maximization of the quality of the diet and the STIR. Therefore, structures between SSH 20 and 23 cm would be within the best spatial distribution and amount of mass in the sward (Fig. 3), while the structures of SSH 14, 17 and 26 cm possibly promoted increasing displacement and search strategies by the animals, resulting in shorter time at feeding stations that lowered herbage intake, reflecting the theory of optimal foraging^45,46^. Different results regarding BR and TJMR as SSH increased were reported by Mezzalira et al.^22^ and Fonseca et al.^28^, who found increasing rates, which can be attributed mainly to the size of the animal, since those two studies were carried out with cattle. According to Illuis et al.^47^ animals of larger size are less constrained by the physical properties of the pasture structure. Black and Kenney^34^ did not observe changes in the rate of jaw movements of sheep in different structures of *Pennisetum clandestinum* Hochst. and perennial ryegrass. For the TB and SSH relationship, our results agree with those of Mezzalira et al.^22^ and Hirata et al.^48^.

The SSH of tall fescue corresponding to the maximum STIR of sheep was 22.3 cm. That SSH can be adopted as an optimal pre-grazing height for rotational stocking systems. Carvalho^29^ suggested using the SSH corresponding to the maximum short-term intake rate as a target for pre-grazing height, with the post-grazing depletion criterion being a 40% decrease in SSH from the pre-grazing SSH^22,28^. He named this management strategy as “Rotatinuous stocking”. Savian^49^, in a long-term experiment, compared “Rotatinuous stocking” with traditional rotational stocking of sheep grazing annual ryegrass pastures and found higher intake rate, higher daily intake, better chemical composition (crude protein, acid detergent fiber, and neutral detergent fiber), higher herbage digestibility and animal performance, and decreasing methane emissions under the “Rotatinuous stocking” management. Following the model of Carvalho^22^ and based on the optimal STIR for tall fescue found in this experiment, suggests a rotational stocking management system of placing animals on tall fescue swards when the pre-grazing SSH is 22.3 cm and removing animals when the SSH declines to 13.4 cm.

## Materials and methods

### Ethics approval

The experimental animals were conducted in accordance with the Guide for the Care and Use of Agricultural Animals in Agricultural Research and Teaching and Directive 2010/63/EU of The European Parliament and of The Council of 22 September 2010 on the protection of animals used for scientific purposes. All procedures involving animals were approved by the Commission for Ethics in the Use of Animals of the Sector of Agricultural Sciences of the Federal University of Paraná (024/2016).

### Experimental site

The experiment was carried out at the Canguiri experimental farm of the Federal University of Paraná—UFPR in Pinhais city, Paraná state, Brazil (25° 26′ 30′′ S and 49° 7′ 30′′ W). The experiment was established in a 3,000 m^2^ experimental area of *Schedonorus arundinaceus* [Schreb.] Dumort cv. INIA Aurora (nomenclature suggested by Soreng et al.^50^, previously named *Festuca arundinacea* Schreb. and tall fescue as common name) sown in June 2015 using conventionally tilled seedbed preparation, with a seeding rate of 55 kg ha^-1^. Beginning September 2015, the experimental area was managed under continuous stocking with sward surface height maintained between 10 and 15 cm, except just prior to and during the grazing events when the different pre-grazing SSH treatments were imposed (see next section).

Nitrogen, phosphorus and potassium were applied uniformly to the experimental area. Before sowing, 540 kg ha^-1^ of P_2_O_5_ was applied and after sowing (between 3 and 5 leaf stage) 200 kg ha^-1^ of N and 60 kg ha^-1^ of K_2_O were applied. In March 2016, 180 kg ha^-1^ of N and 40 kg ha^-1^ of K_2_O were applied. All fertilizer applications were based on the soil chemical analysis done before sowing (depth 0.00 – 0.20 m). The soil test results were: 4.55% organic matter ([organic C × 1.74]/10), pH = 5.70 (CaCl_2_), exchangeable aluminum = 0.00 cmol_c_ dm^-3^, K = 0.11 cmolc dm^-3^, Ca = 5.00 cmol_c_ dm^-3^, Mg = 3.10 cmol_c_ dm^-3^, V (%) = 71 and P = 2.90 mg dm^-3^.

### Treatments and experimental design

Five pre-grazing SSH (14, 17, 20, 23, 26 cm) were evaluated in a randomized complete block design with four replicates. The heights were achieved by allowing regrowth and development of the plants from an initial residue height of 7 cm. The time of day (morning or afternoon) was used as a blocking criterion. Twenty grazing tests of 45 ± 1 min were performed between 24 June and 12 July 2016, with two tests in the morning (between 8:30 and 9:30) and two in the afternoon (between 15:30 and 16:30).

### Sward measurements

To determine the pre- and post-grazing SSH, a sward stick^51^ was used to for 150-point evaluations (≈ 1 point. m^−2^) within each sampling unit. A total of three forage samples of 0.25 m^2^ each per experimental unit were harvested at ground level at pre- and post-grazing, to obtain the total herbage mass (HM), leaf lamina mass (LLM), pseudo-stem + sheath mass (PSM), senescent mass (SM) and other species mass (OSM), determined through morphological and botanical manual assessment.

The herbage mass was quantified in strata of the forage sward by collecting two samples from 0.02 m^2^ randomly distributed per experimental unit and stratifying them from the top of the plants to ground level in every vertical 0.03 m stratum. Those samples were separated into leaf lamina and pseudo-stem + sheath and subsequently the herbage bulk density was calculated by dividing the dry mass by the volume of the sampled cube in each stratum (0.0006 m^3^). The number of green leaves in each stratum was also recorded. All samples were dried in a forced air oven at 65 °C until reaching constant weight for mass measurements.

### Animal measurements

Six White Dorper x Suffolk ewes were used with an average weight of 61.9 ± 5.5 kg and two years of age. Three test animals were used to determine STIR. All animals were previously adapted to the experimental procedure and maintained in an area similar and adjacent to the experimental paddocks.

Before the grazing tests, the animals were equipped with diapers for collecting feces and urine and with IGER (Institute of Grassland and Environmental Research) Behaviour Recorders (Ultra Sound Advice, London, UK)^52^ which record grazing jaw movements and the effective eating time (the length of time that an animal spends eating during grazing). The data were analyzed with the Graze software^53^ and used to calculate bite mass (BM), bite rate (BR), time per bite (TB), total jaw movement rate (TJMR) and effective eating time (ET).

After the 45-min grazing test, the animals were allocated to an adjacent area of 9 m^2^ under open air condition, without access to water and food for 45 min, to estimate insensitive weight losses (H_2_O evaporation, CO_2_ and CH_4_ losses)^54^. The STIR was estimated by the double-weighing technique^55^. A digital balance (MGR-3000 Junior, Toledo, Canoas, Brazil) with a precision of 10 g was used to determine herbage intake. Equation (1) was used for the calculation of STIR:

1 $$STIR=d X \left\{\left[\frac{\left(W2-W1\right) }{(t2-t1)}\right]+\left[\frac{\left(W3-W4\right)}{(t4-t3)}\right]\right\} X \left[\frac{\left(t2-t1\right)}{ET}\right]$$

where *d* is the dry matter content of the herbage; *W1* and *W2* are pre- and post-grazing animal weight respectively; *t1* and *t2* are pre- and post-grazing time; *W3* and *W4* are animal weight pre- and post-insensible weight losses; *t3* and *t4* are pre- and post-insensible loss time and *ET* is effective eating time.

Bite mass was calculated by dividing the herbage intake during the grazing test by the total number of bites. Time per bite was calculated by dividing the total number of bites by ET. Total jaw movement rate was calculated by dividing the total number of jaw movements by the ET during the grazing test. The dry matter content of the herbage was accessed by samples collected by the continuous bite monitoring method^56^, after each grazing test. The fresh herbage mass was weighed after the grazing simulation and then dried in a forced air oven at 65 °C until reaching constant weight.

### Statistical analysis

The paddocks were considered as the experimental units and the animals as sampling units within each paddock. The data set were analyzed using the R software^57^. Data were submitted to analysis of variance (ANOVA). Bulk density data were analyzed with sources of variation being sward surface height, strata and their interaction. When the F-test for treatment differences was significant (*p* < 0.05), the treatment means were compared using the Tukey test, at a significance level of 5%. The grazing variables (STIR, BM, BT, TB, TJMR and ET) were analyzed using a quadratic model (y_*ij*_ = a + bx + cx^2^ + error_*ij*_). The model included SSH as a fixed effect and paddocks and animals within paddocks as random effects. The correlation coefficient (r) was used as a measure of dependence between the variables.
